# Maternal Preconception Body Size and Early Childhood Growth during Prenatal and Postnatal Periods Are Positively Associated with Child-Attained Body Size at Age 6–7 Years: Results from a Follow-up of the PRECONCEPT Trial

**DOI:** 10.1093/jn/nxab004

**Published:** 2021-03-09

**Authors:** Phuong Hong Nguyen, Melissa F Young, Long Quynh Khuong, Lan Mai Tran, Thai Hong Duong, Hoang Cong Nguyen, Reynaldo Martorell, Usha Ramakrishnan

**Affiliations:** International Food Policy Research Institute, Washington, DC, USA; Thai Nguyen University of Pharmacy and Medicine, Thai Nguyen, Vietnam; Emory University, Atlanta, GA, USA; Hanoi University of Public Health, Hanoi, Vietnam; Thai Nguyen National Hospital, Thai Nguyen, Vietnam; Thai Nguyen University of Pharmacy and Medicine, Thai Nguyen, Vietnam; Thai Nguyen National Hospital, Thai Nguyen, Vietnam; Thai Nguyen University of Pharmacy and Medicine, Thai Nguyen, Vietnam; Thai Nguyen National Hospital, Thai Nguyen, Vietnam; Emory University, Atlanta, GA, USA; Emory University, Atlanta, GA, USA

**Keywords:** conditional growth, gestational weight gain, preconception maternal nutritional status, overweight/obese, stunting, Vietnam

## Abstract

**Background:**

Growth faltering is associated with adverse consequences during childhood and later life. However, questions remain on the relative importance of preconception maternal nutritional status (PMNS) and child growth during the first 1000 d of life.

**Objectives:**

We examined associations between PMNS, gestational weight gain (GWG), and child growth during the first 1000 d with attained body size at age 6–7 y.

**Methods:**

We used data from a follow-up of a double-blinded randomized controlled trial of preconception micronutrient supplementation in Vietnam (*n* = 5011 women). The outcomes included offspring height-for-age *z* score (HAZ), BMI-for-age *z* score (BMIZ), and prevalence of stunting and overweight/obese at age 6–7 y (*n* = 1579). We used multivariable linear and Poisson regression models to evaluate the relative contributions of PMNS (height and BMI), GWG, and conditional growth in 4 periods: fetal, 0–6 mo, 6–12 mo, and 12–24 mo.

**Results:**

PMNS was positively associated with child-attained size at 6–7 y. For each 1-SD higher maternal height and BMI, offspring had 0.28-SD and 0.13-SD higher HAZ at 6–7 y, respectively. Higher maternal BMI and GWG were associated with larger child BMIZ (β: 0.29 and 0.10, respectively). Faster linear growth, especially from 6 to 24 mo, had the strongest association with child HAZ at 6–7 y (β: 0.39–0.42), whereas conditional weight measures in all periods were similarly associated with HAZ (β: 0.10–0.15). For BMIZ at 6–7 y, the magnitude of association was larger and increased with child age for conditional weight gain (β: 0.21–0.41) but smaller for conditional length gain. Faster growth in the first 2 y was associated with reduced risk of stunting and thinness but increased risk of overweight/obese at 6–7 y.

**Conclusions:**

Interventions aimed at improving child growth while minimizing the risk of overweight during the school age years should target both women of reproductive age prior to conception through delivery and their offspring during the first 1000 d. The trial was registered at clinicaltrials.gov as NCT01665378.

## Introduction

The first 1000 d of life that begins from conception to the second birthday is a time of rapid development of a child's brain and organ systems and has been identified a critical window of opportunity for influencing child growth and long-term consequences (1, 2). Stunting [length-for-age *z* score, height-for-age *z* score (HAZ) ←2 SD], a population-level indicator of the adequacy of linear growth during this period, has been associated with poor cognition and educational performance, adult height attainment, reduced productivity and lifelong earnings, increased morbidity and mortality, and intergenerational effects (1–5). Given the importance of early linear growth, global targets have been set to reduce child stunting by 40% by 2025 (6). However, many nutrition and health interventions during infancy and the second year of life have had modest or null findings (7–9). To address this public health challenge, there have been calls to better understand the complex and multifactorial influences of child growth patterns, especially the contribution of maternal nutrition before and during pregnancy.

There is growing evidence on the critical role of maternal nutrition for child growth and development (3, 10–13), but less is known about the relative importance of maternal nutrition before and during pregnancy. We have previously reported that preconception nutrition has a similar and independent influence on birth outcomes compared with maternal nutrition during pregnancy using prospective data from a cohort of Vietnamese women (12). Women with a preconception weight <43 kg or a gestational weight gain <8 kg were around 3 times more likely to give birth to a small for gestational age (SGA) or low birth weight infant. Furthermore, women with preconception height <150 cm or a weight <43 kg were at nearly twice the increased risk of having a stunted child at 2 y (13). However, the long-term influence of maternal preconception nutritional status remains largely unknown.

Child growth during early childhood may set the stage for a lifetime (14–16). Evidence from the Consortium on Health Orientated Research in Transitional Societies (COHORTS) collaboration using data from 5 prospective birth cohort studies found that faster linear growth from birth to age 2 y was associated with reduced risk of adult short stature but not risk of overweight (14). In contrast, faster weight gain during the first 2 y of life was associated with increased risk of overweight in adulthood and elevated blood pressure (14). These studies also showed that child growth patterns during early childhood may influence intergenerational offspring birth outcomes (15). Although COHORTS data provide powerful evidence on the importance of early child growth patterns, these studies were carried out >30 y ago and did not examine the role of maternal nutrition both prior to conception and during pregnancy, nor have reliable measures of growth in utero. Those results may not be generalizable in light of the rapid changes that have occurred in the food and built environment in recent decades characterized by increased reliance on unhealthy ultra-processed foods and sedentary lifestyles. Updated research is needed given the emerging nutrition transition and double burden of malnutrition in low-resource settings.

Understanding the patterns and determinants of child growth from conception through the school-age years is critical for designing effective program and policies that address the growing global health challenge of the double burden of malnutrition (i.e., under- and overnutrition that cluster within households and communities in many low- to middle-income countries) (17, 18). This is particularly relevant for Vietnam, a country that is experiencing rapid social and economic change and nutrition transition (19); the prevalence of child (<5 y) stunting has declined (from 40% in early 2000 to 23% in 2017), whereas overweight has increased (from 2.6% to 5.9%) (20). Overweight and obesity are also high among school-age children (at 17% and 8.6%, respectively) (21). Currently, there are limited data from well-designed longitudinal studies that include preconception, pregnancy, and early childhood in low-resource settings to allow for understanding the relative importance of timing across and beyond the first 1000 d. We have the opportunity to use prospectively collected data from the Preconception micronutrient supplementation (PRECONCEPT) study to address these gaps by examining the associations between preconception maternal nutritional status (PMNS), gestational weight gain (GWG), and child growth during the first 1000 d with child malnutrition and attained body size at age 6–7 y.

## Methods

### Data sources and study population

**FIGURE 1:**
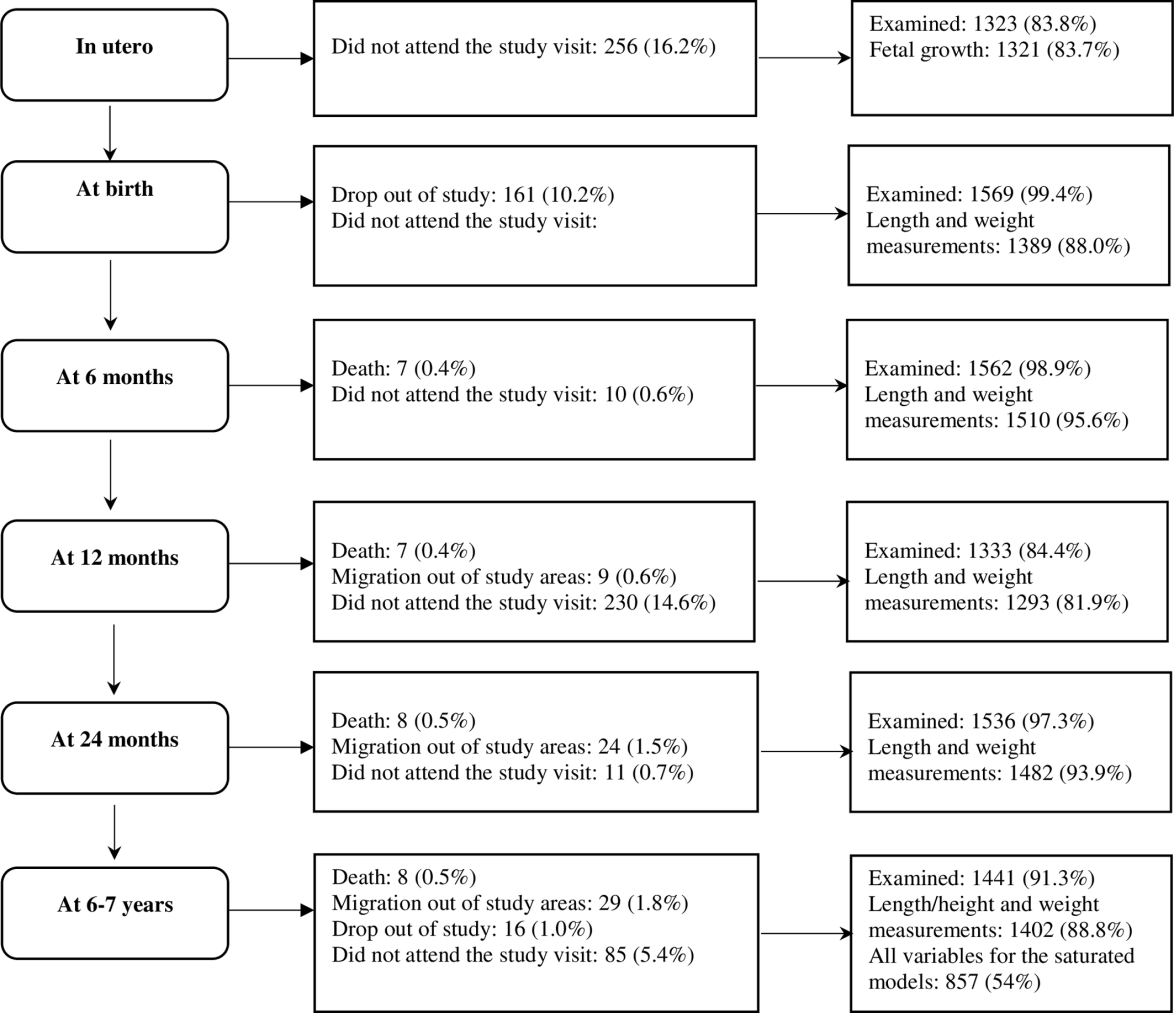
Details of follow-up for the study sample of 1579 singleton livebirths from the first 1000 d to early childhood. All percentages were calculated using the total eligible birth sample (*n* = 1579).

Children in this study are offspring of women who participated in a double-blind randomized controlled trial (PRECONCEPT; registered at clinicaltrials.gov as NCT01665378), which evaluated the effects of preconception micronutrient supplementation on maternal and child health outcomes (22). Details of the parent study have been published previously (22). Briefly, 5011 women of reproductive age were randomly assigned to receive weekly supplements containing 2800 μg folic acid (FA), 60 mg iron and 2800 μg FA (IFA), or multiple micronutrients (MMs, containing the same amount of IFA and 15 micronutrients as shown in **Supplemental Table 1**), from baseline until conception, followed by daily prenatal supplements containing 60 mg iron and 400 μg FA until delivery. Women were followed prospectively to identify pregnancies and evaluate birth outcomes; 1813 women conceived between 2012 and 2014, and 1599 had live births (1579 singleton births, 10 twins). Live births were followed at 6 mo and at 1, 2, and 6–7 y (**Figure 1**) with follow-up rates of 96%, 82%, 94%, and 89%, respectively. We have previously shown that weekly supplementation with MMs or IFA improved linear growth at age 2 y (23), but no impact was found at age 6–7 y (24) compared with FA. The current analysis includes 1402 women who delivered singleton live infants with available data on maternal preconception height/weight and offspring anthropometry data at 6–7 y.

### Outcome measurements

Child weight and height at 6–7 y were measured by trained and standardized field staff using standard methods (25). Child weight was measured using electronic weighing scales precise to 10 g, and child height was measured with collapsible length boards, which were precise to 1 mm. The average of duplicate measurements of height and weight was then converted into HAZs and BMI-for-age *z* scores (BMIZs) according to 2006 WHO child growth standards (26). Stunting and thinness were defined as HAZs and BMIZs below –2 *z* score, respectively. Overweight/obese was defined as BMIZ above 1 *z* score (27, 28).

### Predictor variables

#### Preconception maternal nutritional status and gestational weight gain

Maternal prepregnancy weight and height were measured at enrollment (preconception) in community health centers by trained staff using standard procedures (25, 29). Prepregnancy BMI was calculated as weight/height^2^ (kg/m^2^). Maternal underweight was defined as BMI <18.5 and overweight as BMI >25. Gestational weight gain was calculated from maternal weight measured at delivery and prepregnancy weight. Currently in Vietnam, there are no local weight gain recommendations, and thus we compared gestational weight gain in relation to Institute of Medicine (IOM) recommendations to define those above or below IOM recommendations (30).

#### Offspring growth across the first 1000 d

Offspring growth during the first 1000 d was measured by ultrasound for fetal growth and child anthropometry at birth, 6 mo, 1 y, and 2 y of age. Fetal measurements including head circumference, abdominal circumference, and femur length were obtained during routine prenatal care visits in the second and third trimesters of pregnancy by trained obstetricians, using standardized ultrasound procedures. Details of the ultrasound examination techniques used are provided elsewhere (31). Duplicate measures obtained from separate scans were then averaged and used to estimate fetal weight using the Hadlock formula (32). Fetal weight and femur length were used in estimating conditional weight gain and linear growth, respectively. Birth weight and birth length was measured within 7 d after birth using standard procedures (25, 29). Gestational age was estimated based on the date of last menstrual period that was obtained prospectively by village health workers during their biweekly home visits. Child weight and length at 6 mo, 1 y, and 2 y were collected using standard procedures as described above (25).

Conditional measures of child growth were developed to produce uncorrelated estimates of length and weight gain for the following 4 specific windows during the first 1000 d: *1*) from the second trimester to birth, *2*) birth to <6 mo, *3*) 6 to <12 mo, and *4*) 12–24 mo. Conditional growth is a derived variable and computed as the standardized residuals from linear regressions of anthropometric measures (at a given age) on all prior measures. The conditional length was present length accounting for previous length and weight (but not present weight). Conditional relative weight was present weight accounting for present length and all previous weight and length measures. Conditional growth measures can be interpreted as length/weight deviation from child previous growth trajectory and thus is an indicator of relative speed of length/weight gain/loss within an interval of childhood, independent of child length/weight at a previous time point(s). This method allows for a simultaneous analysis of all growth measures to identify the period of growth most critical to child-attained size at 6–7 y. It has been used increasingly in recent studies to study relations of linear growth and relative height/weight at different age ranges in early childhood with outcomes at later childhood and adulthood (14, 33).

### Potential confounders

Potential confounding variables included maternal age, parity, preconception anemia, child age, sex, preterm status, and household socioeconomic status (SES). Gestational age was calculated as the number of days between the day of delivery and the first day of the last menstrual period, which was obtained prospectively by village health workers during their biweekly home visits. A preterm birth was defined as a birth occurring before 37 completed weeks of pregnancy. Hemoglobin concentrations were measured from capillary blood samples (by finger prick) at preconception using a portable field B-Hemoglobin Analyzer (Radiometer Pacific Pty. Ltd) (34); anemia was defined as a hemoglobin value <12 g/dL (35). Household SES was measured at baseline and was calculated using a principal components analysis of assets and services, including house and land ownership, housing quality, access to services (electricity, gas, water, and sanitation services), and household assets (productive assets, durable goods, animals, and livestock). The first component derived from component scores was used to divide household SES into quartiles (36, 37).

### Statistical analysis

Normality of the continuous outcome variables was assessed using the Kolmogorov–Smirnov test. Descriptive analyses (means, SDs, percentages) were used to report characteristics of the study population. Multivariable linear regressions with robust standard errors were used to examine associations between PMNS indicators, GWG, and child conditional growth in early childhood with child-attained size at 6–7 y (continuous variables). These models were built in 3 steps: *1*) maternal preconception height, BMI, and GWG; *2*) child conditional measures; and 3) both maternal and child measures together. All models adjusted for child (age, sex, and preterm status), maternal (age, parity, and anemia at baseline), and household levels (SES at preconception), as well as treatment group and duration of the preconception intervention. Results for these models were expressed as differences in HAZ and BMIZ associated with a 1–*z* score change in maternal height, BMI, or child conditional variables. We also conducted sensitivity analysis using full-information maximum likelihood for estimation while accounting for missing data among the controlled variables under the assumption of missing at random and without having to do imputation (38). For the dichotomous indicators of child malnutrition at 6–7 y, we used multivariable Poisson regressions, and results were expressed as incident risk ratios. All data analyses were performed using Stata version 16 (StataCorp). Results were considered significant when *P* < 0.05.

### Ethical approval

The study was approved by the Ethical Committee of the Institute of Social and Medicine Studies in Vietnam and the Emory University Institutional Review Board, Atlanta, Georgia, USA. The trial was registered in at clinicaltrials.gov as NCT01665378. Written informed consent was obtained from all study participants.

## Results

The final study sample included the singleton live births born to 1402 women with available data on maternal preconception height/weight and offspring anthropometry data at 6–7 y. On average, mothers were 26 y old at the time of enrollment, and >90% had 1 child. More than half of the mothers had completed middle school (54%), and 38% had completed high school or higher. Of the 1402 women included in the study, 30% had height <150 cm, 25.6% were underweight, and 5.7% were overweight/obese (**Table 1**). More than two-thirds of women had gestational weight gain below the IOM recommendation (30).

**TABLE 1:** Comparison of characteristics of the study sample and measures of offspring size and growth during the first 1000 d through 6–7 y in the final analytic sample and those missing data at follow-up^1^

Characteristic	Study sample^2^ (*n* = 1402)	Missing data at age 6–7 y (*n* = 177)
Maternal characteristics		
Age, y	25.9 ± 4.3	25.8 ± 4.2
Education, %		
Primary school	7.3	14.8
Middle school	55.3	44.3
High school	25.7	22.7
College or higher	11.6	18.2
Improved drinking water, %	90.8	88.6
Improved sanitation facilities, %	96.6	96.0
Prepregnancy weight, kg	45.8 ± 5.5	45.7 ± 5.4
Prepregnancy height, cm	152.7 ± 5.0	152.7 ± 5.3
Prepregnancy BMI, kg/m^2^, %		
<18.5	25.5	69.9
18.5 to <25	68.9	25.5
≥25	5.7	4.6
Gestational weight gain,^3^ kg, %		
Below IOM recommendation	68.9	69.9
Within IOM recommendation	25.5	25.5
Above IOM recommendation	5.7	4.6
Child characteristics		
Female, %	49.5	49.4
Gestational age, wk	39.2 ± 2.0	39.2 ± 2.1
Preterm, %	9.5	13.2
Exclusive breastfeeding at 3 mo, %	59.4	57.5
Adequate diet at 12 mo, %	75.5	70.9
Adequate diet at 24 months, %	58.6	61.3
Attained size and growth measures		
Second to third trimester of pregnancy		
Fetal weight, g	1.4 ± 0.7	1.3 ± 0.8
Femur length, cm	4.9 ± 1.8	4.6 ± 1.5
Birth		
Birth weight, g	3089 ± 444	3044 ± 411
Birth length, cm	49.0 ± 3.0	48.9 ± 3.5
Low birth weight, %	4.7	4.7
SGA, %	15.2	18.0
Stunting, %	11.0	12.7
Overweight/obese, %	9.2	13.4
At 6 mo		
Weight, kg	5.2 ± 0.7	5.1 ± 0.8
Length, cm	57.4 ± 2.8	57.0 ± 2.7
LAZ	−0.1 ± 1.0	−0.2 ± 1.1
BMIZ	−0.1 ± 1.0	0.0 ± 1.0
Stunting, %	3.6	7.6
Overweight/obese, %	12.9	15.9
At 12 mo		
Weight, kg	8.0 ± 1.0	8.0 ± 1.0
Length, cm	68.7 ± 2.5	68.7 ± 2.6
LAZ	−0.6 ± 1.0	−0.5 ± 1.0
BMIZ	−0.1 ± 1.0	−0.1 ± 0.9
Stunting, %	7.2	8.4
Overweight/obese, %	11.9	9.9
At 2 y		
Weight, kg	9.8 ± 1.0	9.6 ± 1.1
Length, cm	78.0 ± 2.7	77.5 ± 3.0
LAZ	−1.1 ± 0.9	−1.2 ± 1.0
BMIZ	−0.0 ± 0.8	−0.1 ± 0.8
Stunting, %	16.3	22.9
Overweight/obese, %	9.6	8.4
At 6–7 y		
Weight, kg	18.8 ± 3.2	
Height, cm	113.6 ± 5.3	
HAZ	−0.8 ± 0.9	
BMIZ	−0.7 ± 1.1	
Stunting, %	9.6	
Overweight/obese, %	6.9	

1 Values are means ± SDs or percentages. BMIZ, BMI-for-age *z* score; HAZ, height-for-age *z* score; IOM, Institute of Medicine; LAZ, length-for-age *z* score; SGA, small for gestational age.

2 Values are numbers (percentages).

3 Currently in Vietnam, there are no local weight gain recommendations, and thus we compared gestational weight gain in relation to IOM recommendations to define those above or below IOM recommendation.

Measures of offspring growth and attained size during the first 1000 d through age 6–7 y are also presented in Table 1. Mean birth weight and length were 3.1 kg and 49 cm, respectively. Stunting declined from 11% at birth to 3.6% at 6 mo, followed by an increase to 16.3% at 2 y, and reduced to 9.6% at age 6–7 y. Overweight/obesity declined from 13% at 6 mo to 7% at 6–7 y.

Maternal preconception height and BMI were positively associated with child-attained size at 6–7 y (**Table 2**). For each 1-SD higher maternal height, offspring had 0.29-SD higher HAZ at 6–7 y. A 1-SD greater maternal BMI was also associated with 0.12-SD and 0.28-SD greater HAZ and BMIZ at 6–7 y, respectively. Gestational weight gain was also positively associated with child BMIZ (β: 0.11). All these associations between maternal anthropometry before and during pregnancy and child size at 6–7 y were attenuated after controlling for measures of child conditional growth during the first 1000 d but remained significant, except for the association between GWG and child BMIZ.

**TABLE 2:** Association of maternal preconception nutrition status and gestational weight gain with child- attained HAZ and BMIZ at 6–7 y^1^

	Model 1^2^		Model 2^3^	
Outcomes	Complete case (*n* = 1271)	FIML^4^ (*n* = 1402)	Complete case (*n* = 857)	FIML (*n* = 1402)
HAZ				
Maternal preconception height	0.29^5^ (0.24, 0.34)	0.28^5^ (0.24, 0.33)	0.07^6^ (0.02, 0.12)	0.09^5^ (0.05, 0.13)
Maternal preconception BMI	0.12^5^ (0.07, 0.17)	0.13^5^ (0.08, 0.18)	0.06^7^ (0.01, 0.11)	0.07^6^ (0.02, 0.11)
Gestational weight gain	0.02 (−0.03, 0.07)	0.03 (−0.02, 0.08)	−0.02 (−0.07, 0.03)	−0.01 (−0.06, 0.03)
BMIZ				
Maternal preconception height	−0.02 (−0.08, 0.04)	−0.02 (−0.08, 0.04)	−0.07^7^ (−0.14, –0.01)	−0.04 (−0.10, 0.01)
Maternal preconception BMI	0.28^5^ (0.21, 0.34)	0.29^5^ (0.23, 0.35)	0.16^5^ (0.10, 0.23)	0.17^5^ (0.12, 0.23)
Gestational weight gain	0.11^5^ (0.05, 0.17)	0.10^5^ (0.04, 0.16)	0.03 (−0.04, 0.09)	0.01 (−0.04, 0.07)

1 Values are βs (95% CIs). BMIZ, BMI-for-age *z* score; FIML, full-information maximum likelihood with missing values; HAZ, height-for-age *z* score.

2 Model 1 used maternal preconception nutrition status and gestational weight gain as main predictors, adjusted for child age, sex, preterm status, mother age, parity, preconception anemia, household socioeconomic status, treatment group, and duration of the preconception intervention.

**FIGURE 2:**
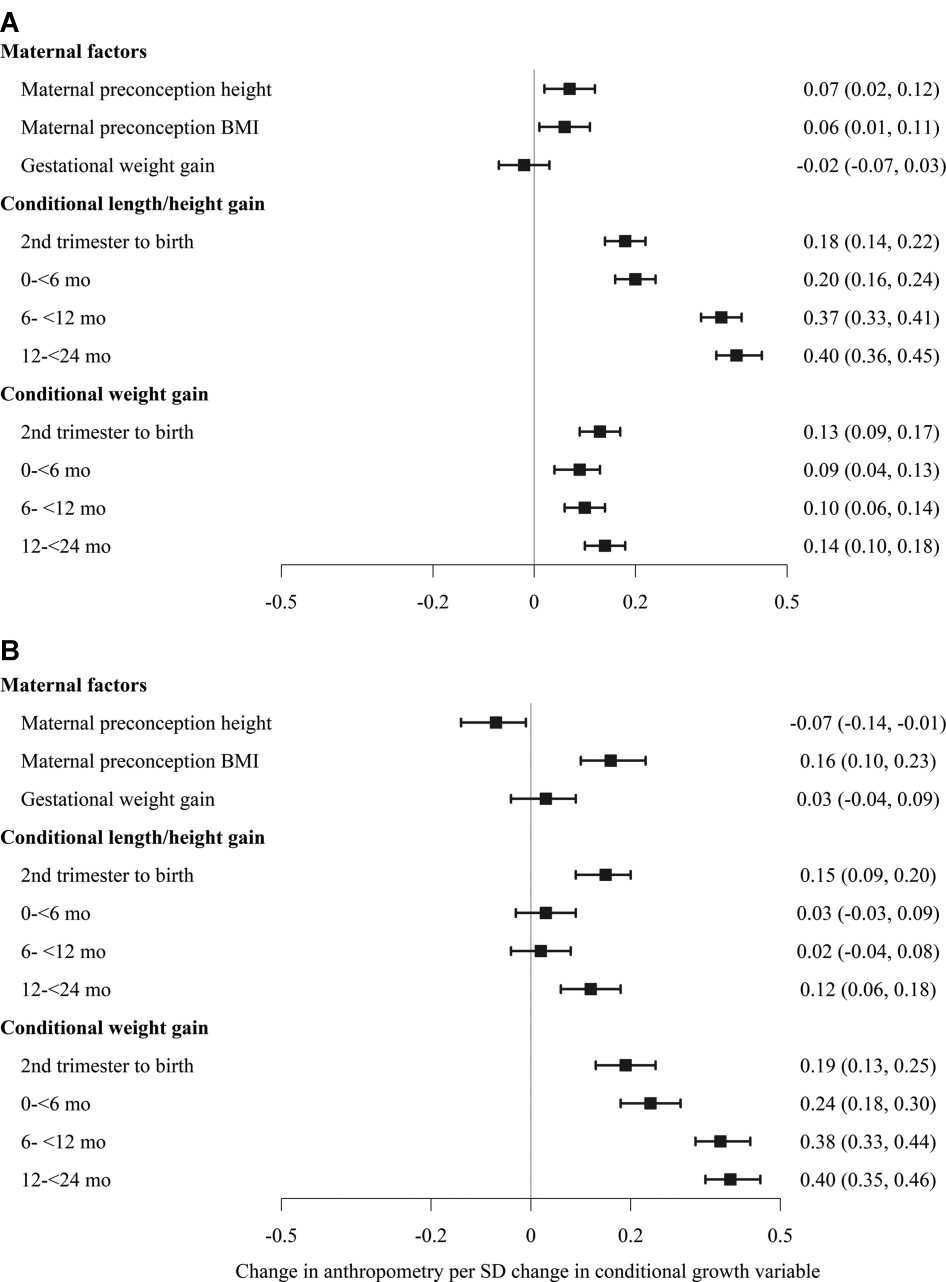
Association of maternal preconception nutrition status and early childhood conditional relative weight gain and height gain with attained HAZ and BMIZ at 6–7 y: (A) HAZ 6–7 y and (B) BMIZ 6–7 y. Values are βs (95% CIs). Results from full model include both maternal and child conditional growth variables, adjusted for child age, sex, preterm status, mother age, parity, preconception BMI and anemia, household socioeconomic status, treatment group, and duration of the preconception intervention. BMIZ, BMI-for-age *z* score; HAZ, height-for-age *z* score.

3 Model 2 included both maternal and child conditional growth variables, adjusted for all covariates as in model 1. Results for the child conditional growth variables are shown in Figure 2.

4 Full-information maximum likelihood for estimation while accounting for missing data among the independent variables under the assumption of missing at random and without having to do imputation.

5 *P* < 0.001.

6 *P* < 0.01.

7 *P* < 0.05.

We also found significant positive associations between conditional relative lengths and weight gain and measures of child attained size at 6–7 y (**Figure 2**), and these relations were robust and remained even after adjustment for maternal variables. For HAZ at 6–7 y, the magnitude of the associations was larger for conditional lengths at 1 and 2 y (β: ∼0.4 SD) compared with those at birth or 6 mo (β: ∼0.2 SD), whereas lower estimates were seen for conditional weight (β: 0.10–0.15 SD). In contrast, estimates for the association between conditional weight gain and BMIZ at 6–7 y were larger (β: 0.19–0.40) and strengthened with increasing age at measurement. Smaller associations were found for conditional length gain in utero (β: 0.14) and from 12 to 24 mo (β: 0.10) with BMIZ at 6–7 y. Sensitivity analysis using full-information maximum likelihood that dealt with missing data showed similar results as in completed case analyses.

Similar findings were found for indicators of child stunting and overweight/obesity. For each SD higher maternal height, the risk of offspring stunting at 6–7 y was reduced by 48% (**Supplemental Table 2**). In addition, a 1-SD greater maternal BMI was associated with reduced child stunting by 29% but with 70% higher child overweight/obesity. Faster growth in the first 1,000 d was also associated with lower prevalence of stunting but higher overweight/obesity at 6–7 y (Supplemental Table 2 and **Supplemental Table 3**). The risk of stunting reduced by 29–55% for each 1–*z* score change in the conditional length and by 16–32% for each 1–*z* score change in the conditional weight. In contrast, the risk for overweight/obesity increased by 1.3–1.8 times for each 1–*z* score change in the conditional weight. Sensitivity analysis that restricted the sample to term births also revealed similar results (data not shown).

## Discussion

Using prospectively collected data from preconception through age 6–7 y, our findings demonstrate the importance of PMNS, GWG, and early child growth during the first 1000 d on child-attained size and risk of malnutrition among young school-aged children at 6–7 y. Prepregnancy height was positively associated with child HAZ at 6–7 y of age, and these associations remained even after accounting for GWG and child growth in the first 2 y. We also found that linear growth conditionals (particularly from 6 to 24 mo) had the strongest association with HAZ at 6–7 y (effect size: 0.4 SD), whereas weight conditional measures mattered little (effect size: 0.10–0.15 SD). For BMIZ, the opposite is the case, where the association was most pronounced with weight conditional measures, together with preconception maternal BMI.

Several studies, mostly observational and from developed countries, have demonstrated the importance of maternal nutritional status prior to conception for reproductive health outcomes and offspring growth, development, and long-term health (39, 40). Overweight women are especially at increased risk of preeclampsia and delivering preterm, whereas underweight women are at increased risk of delivering SGA infants, who in turn are more likely to experience subsequent growth failure and stunting by age 2 y (11). Possible mechanisms that can explain the association between maternal anthropometry prior to conception and offspring birth weight include shared genes, environment, and epigenetic changes (15). Consistent with a growing body of research, our group has also demonstrated the importance of maternal prepregnancy weight for weight gain during pregnancy as well as birth size (12) and offspring linear growth across the first 1000 d (13). In this article, we extend these findings to growth during the first 6–7 y of age using prospectively collected data that allow us to examine the independent contribution of measures of maternal nutrition status both before and during pregnancy after accounting for growth during the first 1000 d on attained size and BMI as children enter school. We found that higher maternal preconception height was associated with higher child HAZ and reduced risk of stunting at 6–7 y but not risk overweight. In contrast, a 1-SD increase in maternal preconception BMI was associated with 1.5 times higher risk of overweight during early childhood. Our findings highlight the need to improve women's height to prevent stunting in the next generation while addressing the need to ensure healthy weight for women in their reproductive years. The preconception period has been recommended as a period of special opportunity for intervention due to its relevance on life course epidemiology, embryo programming around the time of conception, maternal motivation, and disappointment with interventions starting in pregnancy (40). Although preconceptional interventions have the potential to ensure optimal nutritional status (especially weight and micronutrient status), interventions to improve height will require investments much earlier in the life course (7).

Several studies have examined the importance of GWG for maternal and infant outcomes. Inadequate GWG has been associated with increased risk of giving birth to an SGA infant (12), whereas excessive GWG has been associated with greater postpartum weight retention (41–43) and increased risk of child obesity (44). However, the current recommendations for GWG are based primarily on studies from developed countries. Studies from Asia have shown that 31% of women gained below the IOM recommendations and 37% above the recommended GWG guidelines (45), whereas 70% of women in our study had inadequate GWG, and only 6% gained above the recommendation. Although the current study did not find strong associations between GWG and child nutrition status, further research is needed on both the long-term implications of GWG and appropriateness of IOM recommendations, especially in countries experiencing nutrition transition.

Consistent with a growing body of research that has examined the contribution of child growth during the first 1000 d and thereafter to final attained size, our results show that faster linear growth, particularly during between 6 and 24 mo, was associated with child HAZ at 6–7 y (β: 0.41–0.42), even after accounting for the strong effects of preconception maternal height and BMI. Similar growth trajectories by 6 mo of age were also observed in an Australian birth cohort study (46). The more pronounced results after 6 mo suggest that we need to continue to pay attention to the adequacy of feeding practices and the environment during early childhood (47, 48). The COHORTS studies demonstrated the importance of growth during the first 2 y of life on adult stature (14), but they do not allow us to examine what happens in the middle period—specifically, preadolescence. These studies also have limited and poor-quality data on maternal nutritional status prior to conception and/or in utero growth.

Our findings confirm previously reported positive associations between faster weight gain in early life with higher BMIZ and higher risks of overweight/obesity at 6–7 y. This is especially relevant in light of the double burden of malnutrition in many low- to middle-income countries where underweight and stunting remain a persistent problem, but overweight and obesity are on the rise (3, 49). Although underweight and stunting among children are associated with increased risk of infectious disease, poor cognitive development, reduced earning potential, and long-term chronic disease risk (3), overweight and obesity among children are also associated with an increased risk of overweight and obesity and cardiometabolic diseases in later life (50–52). Countries such as Vietnam that have or are experiencing rapid changes in nutrition outcomes (53–57) need critical information on how to optimize targeted interventions that will reduce the burden of undernutrition yet prevent overweight/obesity.

Key strengths of our study include a well-designed longitudinal study that includes preconception, pregnancy, and early childhood in low-resource settings with a high follow-up rate of 91% at the age of 6 y. The rich data on maternal preconception, gestational weight gain, and fetal growth, as well as multiple assessments of growth at different ages with a standardized methodology, allowed us to examine the relative importance of timing across and beyond the first 1000 d on child growth at school age. Although birth size measurements were collected up to a week, they were obtained for >90% of the births within 24 h of birth, and sensitivity analysis confirmed the robustness of our conclusions. The use of conditional growth analyses had the advantage of reducing the correlations between multiple measurements and allowed us to separate the roles of linear growth from soft tissue gain (fat mass and fat-free mass) (14). Finally, our outcomes measured at age 6–7 y are useful to represent the beginning of the school age years when children are exposed to new and challenging environments.

This study has some limitations—notably, adequacy of data on energy expenditure and changes in diet during the preschool years. The low prevalence of overweight/obesity among children (at 7%) further limited our power to detect significant associations. Our study was restricted to measures of maternal body size to assess maternal nutritional status, and future work should consider inclusion of additional measures of body composition and nutritional biomarkers. Future studies that continue to follow up this cohort have the potential to advance current understanding of the mechanism and patterns of child growth on intellectual development, cardiometabolic risk, and human capital outcomes later in life.

## Conclusion

Our results suggest important longstanding effects of preconception nutritional status on child growth. This work has implications for prioritizing women's nutrition in low-resource settings to optimize child growth. Balanced multidisciplinary programs and policies that combine nutrition education with family planning services as part of preconception care are needed to ensure optimal maternal preconception BMI and GWG in the efforts to prevent and control for the emerging double burden of nutrition.

## Supplementary Material

nxab004_Supplemental_File

## Notes

Funding for this research was provided by the Nestle Foundation, the Mathile Institute for the Advancement of Human Nutrition, and the Micronutrient Initiative.

Author disclosures: The authors report no conflicts of interest.

Supplemental Tables 1–3 are available from the “Online Supporting Material” link in the online posting of the article and from the same link in the online table of contents at jn.nutrition.org.

Abbreviations used: BMIZ, BMI-for-age *z* score; FA, folic acid; GWG, gestational weight gain; HAZ, height-for-age *z* score; IFA, iron and folic acid; IOM, Institute of Medicine; MM, multiple micronutrient; PMNS, preconception maternal nutritional status; SES, socioeconomic status; SGA, small for gestational age.
