# Supplementary material for: Maternal Preconception Body Size and Early Childhood Growth during Prenatal and Postnatal Periods Are Positively Associated with Child-Attained Body Size at Age 6–7 Years: Results from a Follow-up of the PRECONCEPT Trial
Source: J Nutr. 2021 Mar 9;151(5):1302–10. doi: 10.1093/jn/nxab004 (PMC8112760; doi:10.1093/jn/nxab004)
Supplement: nxab004_Supplemental_File [file nxab004_supplemental_file.docx]

**Supplemental Table 1: The composition of preconception micronutrient supplements**

| **Ingredient** | **Pre-pregnancy (weekly)** | | | **RDA for non-pregnant women^1^** |
| --- | --- | --- | --- | --- |
|  | **MM** | **IFA** | **FA** |  |
| Vitamin A, μg | 800 |  |  | 700 |
| Vitamin D, IU | 600 |  |  | 15 |
| Vitamin E, mg | 10 |  |  | 15 |
| Vitamin C, mg | 70 |  |  | 75 |
| Thiamine, mg | 1.4 |  |  | 1.1 |
| Riboflavin, mg | 1.4 |  |  | 1.1 |
| Niacin, mg | 18 |  |  | 14 |
| Vitamin B_6,_ mg | 1.9 |  |  | 1.3 |
| Vitamin B_12,_ μg | 2.6 |  |  | 2.4 |
| Folic acid, μg | 2800 | 2800 | 2800 | 400 |
| Iron (ferrous sulfate), mg | 60 | 60 |  | 18 |
| Zinc (sulfate), mg | 15 |  |  | 8 |
| Copper, mg | 2 |  |  | 0.9 |
| Selenium, μg | 65 |  |  | 55 |
| Iodine, μg | 150 |  |  | 150 |

^1^IOM. Dietary Reference Intakes (DRIs): Recommended Intakes for Individuals. Food and Nutrition Board, Institute of Medicine, National Academies, 2004. FA: Folic Acid, IFA: Iron and Folic Acid, MM: Multiple Micronutrient, RDA- Recommended Daily Allowance

**Supplemental Table 2: Association of maternal preconception nutrition status and gestational weight gain with child stunting at 6-7y^1^**

|  | **Model 1^2^** | **Model 2^3^** | **Model 3^4^** |
| --- | --- | --- | --- |
|  | ***n*= 1,271** | ***n*= 915** | ***n*= 857** |
|  | **IRR (95% CI)** | **IRR (95% CI)** | **IRR (95% CI)** |
| **Maternal** |  |  |  |
| Maternal preconception height | 0.52^***5^ (0.45,0.62) |  | 0.74^**^ (0.60,0.91) |
| Maternal preconception BMI | 0.71^**^ (0.58,0.87) |  | 0.90 (0.70,1.16) |
| Gestational weight gain | 0.98 (0.83,1.16) |  | 1.10 (0.86,1.41) |
| **Conditional length/ height gain** |  |  |  |
| 2nd trimester to birth |  | 0.66^***^ (0.56,0.78) | 0.71^***^ (0.59,0.85) |
| 0-<6 mo |  | 0.65^***^ (0.53,0.79) | 0.69^***^ (0.57,0.83) |
| 6- <12 mo |  | 0.40^***^ (0.33,0.49) | 0.45^***^ (0.36,0.55) |
| 12-<24 m |  | 0.43^***^ (0.37,0.51) | 0.45^***^ (0.38,0.54) |
| **Conditional weight gain** |  |  |  |
| 2nd trimester to birth |  | 0.83^**^ (0.72,0.95) | 0.84^*^ (0.72,0.99) |
| 0-<6 mo |  | 0.71^**^ (0.57,0.89) | 0.68^***^ (0.54,0.85) |
| 6- <12 mo |  | 0.75^**^ (0.61,0.92) | 0.74^**^ (0.60,0.91) |
| 12-<24 m |  | 0.71^**^ (0.57,0.90) | 0.72^**^ (0.56,0.91) |

^1^Values are IRR (95% CI); ^2^Model 1 used maternal preconception nutrition status and gestational weight gain as main predictors, adjusted for child age, sex, preterm status, mother age, parity, preconception anemia, household SES, treatment group and duration of the preconception intervention. ^3^Model 2 used child conditional growth variables (a derived variable and computed as the standardized residuals from linear regressions of anthropometric measures at a given age on all prior measures) in the first 1,000 days as main predictors, adjusted for all covariate as model 1. ^4^Model 3 included both maternal and child conditional growth variables, adjusted for all covariate as model 1.

^5^Signiﬁcantly different: ****p*<0.001, ***p*<0.01, **p*<0.05. BMI: body mass index-for-age; CI: confident interval; IRR: incidence-rate ratio.

**Supplemental Table 3: Association of maternal preconception nutrition status and gestational weight gain with child overweight/obese at 6-7y^1^**

|  | **Model 1^2^** | **Model 2^3^** | **Model 3^4^** |
| --- | --- | --- | --- |
|  | ***n*= 1,271** | ***n*= 915** | ***n*= 857** |
|  | **IRR (95% CI)** | **IRR (95% CI)** | **IRR (95% CI)** |
| **Maternal** |  |  |  |
| Maternal preconception height | 1.06 (0.86,1.31) |  | 0.87 (0.67,1.12) |
| Maternal preconception BMI | 1.67^***5^ (1.39,2.01) |  | 1.48^*^ (1.09,2.02) |
| Gestational weight gain | 1.10 (0.92,1.32) |  | 1.01 (0.78,1.31) |
| **Conditional length/ height gain** |  |  |  |
| 2nd trimester to birth |  | 1.37^*^ (1.07,1.76) | 1.32 (1.00,1.74) |
| 0-<6 mo |  | 1.00 (0.83,1.20) | 1.07 (0.88,1.28) |
| 6- <12 mo |  | 0.86 (0.67,1.11) | 0.93 (0.69,1.26) |
| 12-<24 m |  | 1.36^**^ (1.10,1.68) | 1.43^**^ (1.12,1.84) |
| **Conditional weight gain** |  |  |  |
| 2nd trimester to birth |  | 1.28^*^ (1.01,1.62) | 1.25 (0.96,1.64) |
| 0-<6 mo |  | 1.33^*^ (1.06,1.67) | 1.35^*^ (1.06,1.72) |
| 6- <12 mo |  | 1.42^**^ (1.12,1.81) | 1.40^**^ (1.09,1.81) |
| 12-<24 m |  | 1.79^***^ (1.48,2.16) | 1.79^***^ (1.47,2.19) |

^1^Values are IRR (95% CI); ^2^Model 1 used maternal preconception nutrition status and gestational weight gain as main predictors, adjusted for child age, sex, preterm status, mother age, parity, preconception anemia, household SES, treatment group and duration of the preconception intervention. ^3^Model 2 used child conditional growth variables (a derived variable and computed as the standardized residuals from linear regressions of anthropometric measures at a given age on all prior measures) in the first 1,000 days as main predictors, adjusted for all covariate as model 1. ^4^Model 3 included both maternal and child conditional growth variables, adjusted for all covariate as model 1.

^5^Signiﬁcantly different: ****p*<0.001, ***p*<0.01, **p*<0.05. BMI: body mass index-for-age; CI: confident interval; IRR: incidence-rate ratio.
